# An Overview of the Evidence and Mechanism of Drug–Herb Interactions Between Propolis and Pharmaceutical Drugs

**DOI:** 10.3389/fphar.2022.876183

**Published:** 2022-04-04

**Authors:** Sanowar Hossain, Muhammad Yousaf, Yang Liu, Dennis Chang, Xian Zhou

**Affiliations:** ^1^ Department of Pharmacy, Pabna University of Science and Technology, Pabna, Bangladesh; ^2^ Department of Bioinformatics and Biotechnology, Government College University, Faisalabad, Pakistan; ^3^ NICM Health Research Institute, Western Sydney University, Penrith, NSW, Australia

**Keywords:** drug-herb interaction, synergy, propolis, chemotherapy, antimicrobial, metformin, CYP450

## Abstract

With the growing interest in the medicinal use of propolis, numerous studies have reported significant interactions between propolis extract and pharmaceutical drugs which may result in great clinical benefits or risks. The present study aims to review the drug–herb interactions of the full-spectrum propolis extract and main pharmaceutical drugs from the pharmacodynamic and pharmacokinetic aspects and elucidate the underlying pharmacological mechanisms. A literature search was conducted between June 2021 and February 2022 in Google Scholar, PubMed, MEDLINE, and EMBASE databases to include English studies from years 2000 to 2022 that evaluated the interaction of full-spectrum propolis extract and standard pharmaceutical drugs/cytochromes P450s. Studies that looked into geopropolis, propolis fractions, and isolated compounds, or interaction of propolis with foods, bioactive molecules, or receptors other than standard pharmaceutical drugs were excluded. From a pharmacodynamic perspective, propolis extract exhibited positive or synergistic interaction with several chemotherapeutic drugs by enhancing antitumor activity, sensitizing the chemoresistance cell lines, and attenuating multi-organ toxicity. The molecular mechanisms were associated with upregulating the apoptotic signal and immunomodulatory activity and attenuating oxidative damage. Propolis extract also enhanced the anti-bacterial and antifungal activities of many antimicrobial drugs against sensitive and resistant organisms, with an effect against the gram-positive bacteria stronger than that of the gram-negative bacteria. The synergistic action was related to strengthened action on interfering cell wall integrity and protein synthesis. The strong antioxidant activity of propolis also strengthened the therapeutic effect of metformin in attenuating hyperglycemia and pancreatic damage, as well as mitigating oxidative stress in the liver, kidney, and testis. In addition, propolis showed a potential capacity to enhance short-term and long-term memory function together with donepezil and improve motor function with levodopa and parasite killing activity with praziquantel. Pharmacokinetic studies showed inhibitory activities of propolis extracts on several CYP450 enzymes *in vitro* and *in vivo*. However, the effects on those CYP450 were deemed insignificant in humans, which may be attributed to the low bioavailability of the contributing bioactive compounds when administered in the body. The enhanced bioactivities of propolis and main pharmaceutical drugs support using propolis in integrative medicine in anti-cancer, anti-microbial, antidiabetic, and neurological disorders, with a low risk of altered pharmacokinetic activities.

## Introduction

The practice of medicinal plants has augmented enormously over the past three decades, with approximately 80% of the population worldwide relying on natural products, including medicinal plants, for primary healthcare (Zhou, Li, 2019; Qadir et al., 2021). The international market of medicinal plants was estimated at US$138,350 million in 2020 and is predictable to reach US$218,940 million by the end of 2026, rising at a rate of 6.7% during 2021–2026 (Market Watch, 2020).

Most individuals consume medicinal plants as part of their cultural belief and consider that the products are safe and have long-lasting efficacy (Ibrahim et al., 2016). Regarding the rising demand and practice of medicinal plants in the general public, wide-ranging research regarding their efficacy and safety when used with conventional medicines is essential (Zhang, Onakpoya, 2015). Indeed, medicinal plants are frequently administered in combination with pharmaceutical drugs without the prescription of general practitioners. This raises concerns of drug–herb interactions as there have been numerous clinical observations on significant adverse reactions (De Smet, 2007; Colalto, 2010). *Hypericum perforatum* L. (St. John’s wort) is known to have significant interactions with numerous pharmaceutical drugs (i.e., antiepileptics, antidepressants, and lipid-lowering drugs) that caused life-threatening events (Soleymani, Bahramsoltani, 2017). *Salvia miltiorrhiza* Bunge was found to exaggerate the anti-coagulant response combined with warfarin (Chan, 2001). *Ginkgo biloba* L. was reported to interact with ibuprofen resulting in deadly intracerebral mass bleeding (Meisel, Johne, 2003). In contrast, drug–herb interactions could be therapeutically valuable in a synergistic manner to enhance the therapeutic efficacy and/or to reduce side effects (Fasinu, Bouic, 2012; Gerber, Steyn, 2018). For instance, co-administration of *Allium sativum* L. (garlic) tablets and metformin was reported to further reduce higher blood glucose levels when compared with the placebo and metformin monotherapy group (Ashraf, Khan, 2011). Thus, it is of great clinical significance to investigate the drug–herb interaction, which may induce adverse consequences or help achieve a more advantageous clinical outcome (Zhou, Fu, 2021).

Drug–herb interactions can occur on both a pharmacodynamic and pharmacokinetics basis. Drug interaction in pharmacodynamics refers to a change of drug action on the target site. This interaction may be a synergistic, additive, or antagonistic effect at the same or different biomarker, receptors, or pathways (Izzo, 2012; Zhou X et al., 2016). Pharmacokinetic interaction involves the modulation of absorption, distribution, metabolism, and excretion of drugs by affecting drug transporters [i.e., p-glycoprotein (p-gp)] and cytochromes P450 (CYP450) enzymes. A pharmacokinetic interaction may rise concern when the modification occurs in drug’s pharmacokinetic parameters [i.e., area under the curve (AUC), the time to maximum plasma concentration (Tmax), or maximum plasma concentration (Cmax)], leading to toxic or adverse effects attributed to an overdose, particularly for those drugs with narrow therapeutic indices (e.g., warfarin, phenytoin, and digoxin) (Tarirai, Viljoen, 2010).

Propolis is a natural resinous material produced by honey bees with mixed saliva and beeswax, along with substances obtained from different parts of plants such as bark, buds, and exudates (da Silveira, Fernandes, 2016). It has been extensively used as a traditional medicine for various ailments for thousands of years (Salatino, Fernandes-Silva, 2011). Nowadays, propolis has gained great popularity as a valuable alternative and complementary medicine attributed to its potent and diverse bioactivities (Osés, Marcos, 2020). Although the chemical composition of propolis depends sufficiently on the specificity of local flora, species of honey bees, climatic and geographical factors, collecting seasons, and plant resources (Ristivojević, Trifković, 2015), a typical resinous mixture of propolis is composed of 40%–70% balsam (phenolic acids and flavonoids), 20%–35% waxes, 1%–3% aromatic and essential oils, and 5% other constituents such as vitamins, minerals, proteins, and enzymes (Huang, Zhang, 2014). The wide range of propolis application in modern medicine is mainly attributed to phenolic acids and flavonoids, which exhibited broad-spectrum biological and pharmacological activities, including antioxidant (Nna et al., 2018a; Gao, Pu, 2018), anti-inflammatory (Bueno-Silva, Rosalen, 2017; Jin, Wang, 2017), anti-hyperglycemic (El Rabey, Al-Seeni, 2017; Samadi, Mozaffari-Khosravi, 2017), immunomodulatory (Orsatti, Missima, 2010), anti-apoptotic (Alm-Eldeen, Basyony, 2017), antifungal (Silici, Koç, 2005), antibacterial (Grecka, Kuś, 2019), and anti-cancer properties (Bhuyan, Alsherbiny, 2021).

The diverse chemical components and pharmacological properties of propolis highlight the possibility of the interaction with many pharmaceutical drugs. Herein, we aimed to conduct a comprehensive review of the drug–herb interaction between full-spectrum propolis extract and pharmaceutical drugs in both pharmacodynamic and pharmacokinetic aspects and elucidate the underlying pharmacological mechanisms.

## Methods

A comprehensive search was conducted between June 2021 and February 2022 of peer-reviewed English journal articles related to propolis herb-drug interaction in PubMed, Google Scholar, Web of Science, EMBASE, and Scopus to collect studies published between 1 January 2000 and December 2021.

The search terms for the review addressed four components. We used “ropolis,” “bee glue,” and “glue, bee” for the keyword “propolis.” The search terms relating to pharmaceutical drugs included “Western drugs,” “conventional drugs,” “traditional drugs,” “conventional medicine,” “western medicine,” “pharmaceutical drugs,” “synthetic drugs,” and “drugs.” The search term “interaction” was enhanced with synonyms and related terms including “interact,” “interaction,” “combine,” “combination,” “compatible,” “formulate,” “formulation,” “synergistic,” “synergism,” “synergize,” “synergise,” “enhance,” “promote,” “augment,” “improve,” “magnify,” “toxify,” and “impair.” We used “cytochrome 450” or “CYP450” when searching for the interaction between propolis and CYP450 enzymes. The identified abstracts from the electronic search were independently reviewed by two authors (SH and XZ) for a further selection of the studies.

We included original research articles on full-spectrum propolis interaction with standard pharmaceutical medicines/CYP450 showing the interaction from pharmacodynamic, pharmacokinetic, and clinical studies. The interaction can be manifested as the comparison of combined effects to individual effects as outcome measurement, elucidation of the underlying mechanistic actions, and altered pharmacokinetic parameters, including CYP450 activities. Articles were also identified through the reference list of retrieved research articles and reviews and specific searching with the name of pharmaceutical drugs. Only articles in English were included. Original studies that looked into geopropolis, honey, propolis fractions, and isolated compounds, or interaction of propolis with foods, bioactive molecules (excluding CYP450), or receptors other than standard pharmaceutical drugs were excluded. Studies that looked into the intervention of propolis only without combining with pharmaceutical drugs were excluded. The combinations that involved ingredient(s) in addition to the propolis and the pharmaceutical drug were excluded. Original research articles that investigated combined therapy of propolis and pharmaceutical drugs, without any comparison to either monotherapies or elucidation of interaction, were excluded.

All the included studies were listed in the EndNote library (XZ), and the recruitment of studies was confirmed independently by a second author (SH). The search strategy has led to 149 studies identified through database searching, and 73 studies were excluded due to the irrelevance of the scope of studies (i.e., studies on geopropolis, honey, and active fractions/compounds in propolis). Then, another eight studies were excluded with reasons such as lack of the elucidation of interaction, propolis monotherapy only, and three or more ingredients used in the combination. Finally, 68 studies were included in this review for evaluation and discussion. Data items include author, year of publication, propolis, dose or ratio, type of study, key findings, and mechanism of interaction, and the methods used to determine interaction were summarized for each paper and narratively described. The flowchart of the study selection process is listed in Supplementary Figure S1.

## Pharmacodynamic Interactions

Some preclinical studies have investigated the pharmacodynamic interaction of propolis extracts with pharmaceutical drugs with particular focuses on anti-cancer, anti-diabetic, anti-microbial, anti-parasitic, and neuroprotective therapies. Positive or synergistic enhanced therapeutic outcomes *via* modulating multiple cellular signaling pathways were largely reported by the following studies.

### Anti-Cancer Drugs

Strong preclinical evidence suggested positive interactions of propolis extracts sourced from various geographical locations and under various preparations in combination with anti-cancer drugs, including doxorubicin (DOX), temozolomide (TMZ), 5-fluorouracil (5-FU), mitomycin C (MMC), irinotecan, and photodynamic therapy (PDT). The positive interaction was manifested as enhanced efficacy, reduced side effects, and/or drug resistance *via* diverse mechanistic actions.

#### Interaction With DOX

DOX is a cytotoxic anthracycline that is the first-line chemotherapy for breast cancer. DOX generates cytotoxic activity mainly attributed to inhibiting topoisomerase II mediated DNA repair to prevent DNA replication and producing free radical damage to DNA (Thorn, Oshiro, 2011). However, the cytotoxicity and oxidative stress induced by DOX also cause significant side effects on multiple organs, and thus it is often used in combination with other medications to lower the dosage and toxicity. Numerous studies have shown the great potential of combining propolis with DOX as a more advantageous therapy aiming to enhance the anti-cancer activity, reduce the chemoresistance, and ameliorate the significant side effects from DOX.

Two *in vitro* studies suggested synergistically enhanced anti-cancer activities of propolis used together with DOX on breast cancer cell lines (Alsherbiny, Bhuyan, 2021; Rouibah et al., 2021). Rouibah et al. (2021) investigated the anti-cancer activity of 70% ethanolic Algerian propolis extract (30 μg/ml) and various concentrations of DOX (0.1–100 μM) on MDA-MB-231 breast cancer cells and suggested that the combination exhibited greater cell growth inhibition as evidenced by a significantly lower IC_50_ value (tenfold lower than using DOX alone). The combination induced the cell cycle arrest in the S phase and caspase-dependent apoptosis. A synergistic anti-cancer activity was also observed in the study from Alsherbiny et al. (2021) who evaluated the combination of ethanolic Australian propolis extract (20–180 μg/ml) and DOX (0.06–0.52 μg/ml) in MCF7 breast adenocarcinoma cells. Using combination index (CI) model and the DrugComb portal, their result demonstrated a strong synergistic interaction of propolis extract and DOX in the ratio of 100:0.29 (*w/w*) in inhibiting cell proliferation. The molecular mechanism for the synergistic interaction may be associated with 1) promoting apoptosis by the regulation of a series of pro-apoptotic (p27, PON2, and catalase) and anti-apoptotic proteins (XIAP, HSP60, and HIF-1α) and 2) anti-oxidant profile of propolis resulting in antioxidant-related apoptotic pathways. In addition, propolis reversed DOX-induced necrosis to programmed cell apoptosis, which may contribute to a reduced cytotoxicity. The shotgun proteomics study suggested 21 significantly dysregulated proteins by the combination compared to the monotreatments, which were associated with the propolis metabolites in the cancer cells. The expressions of these proteins maybe involved in the observed synergistic anti-cancer activity (Alsherbiny et al., 2021).

Although DOX is considered the most effective chemotherapy, drug resistance is often shown in clinics resulting in poor patient prognosis and survival. The main mechanisms associated with the drug resistance of DOX included the diminished action in inducing cell apoptosis mediated by the MAPK/ERK pathway (Christowitz, Davis, 2019) and the overexpression of drug resistance genes, p-glycoprotein, which hindered the penetration of DOX into the nucleus. Remarkably, Rouibah et al. (2021)suggested that propolis inhibited the expression of P-gp in breast cancer cells, and thus it may contribute to the enhanced anti-cancer activity observed in the DOX-propolis combination *via* an increased nuclear permeability of DOX.

DOX is linked with a series of adverse effects, including myocardial toxicity (Ali et al., 2020), nephrotoxicity (Ali et al., 2020), neurotoxicity (Alhowail, Bloemer, 2019), and hepatotoxicity (Singla, Kumar, 2014; Omar, Allithy, 2016), mainly attributed to its actions of DNA damage and generation of free radicals (Pugazhendhi, Edison, 2018). Due to the powerful anti-oxidant activity of propolis extracts (Zabaiou, Fouache, 2017), a few studies investigated the potential capacity of propolis in potentiating the toxicity of DOX when used together. Ali et al. (2020) showed that the 4-week treatment of propolis extract (200 mg/kg/day, gastric intubation) significantly ameliorated DOX (10 mg/kg, i.p.) induced cardiotoxicity in rats. The elevated cardiac biomarkers such as brain natriuretic peptide (BNP), troponin T, lactate dehydrogenase (LDH), creatine kinase (CK), and aspartate aminotransferase (AST) were reduced, and the cardiac oxidation was improved by decreased malondialdehyde (MDA) and upregulated antioxidant enzymes, including catalase, glutathione (GSH), and superoxide dismutase (SOD) in the combined treatment group (Ali et al., 2020). Moreover, Ali et al. (2020) showed that the propolis ameliorated the elevated levels of creatinine and urea against DOX-induced nephrotoxicity in rats (Ali et al., 2020). Ethanolic Egyptian propolis (200 mg/kg, p.o.) treated for 3 weeks restored the testicular function when co-administered with DOX (18 mg/kg, i.p.), in which the protective action was associated with reduced oxidative stress and inflammatory and apoptotic markers (Rizk et al., 2014). In addition, another two *in vivo* studies suggested that the accumulative administration of propolis extract protected liver against the toxicity of DOX in rats (Singla et al., 2014; Omar et al., 2016). Mohamed et al. (2021) suggested that the oral administered propolis (100 mg/kg once daily for 28 days) significantly ameliorated DOX-induced myocardium, liver, kidney, and lung tissues as manifested by reduced injury markers, apoptosis, and pro-inflammatory cytokines (Mohamed, Osman, 2021). Noticeably, most studies agreed that the capacity of propolis in scavenging free radicals [i.e., reactive oxygen species (ROS)] and improving oxidative status plays a key role in the protective activity against DOX (Benguedouar, Boussenane, 2008; Rizk et al., 2014; Singla et al., 2014; Mohamed et al., 2021). Tavares et al. (2007) further explained that propolis significantly decreased the frequency of chromosome damage induced by DOX compared to that of DOX only, which may partially contribute to the capacity of propolis capturing free radicals produced by DOX (Tavares, Lira, 2007).

**FIGURE 1:**
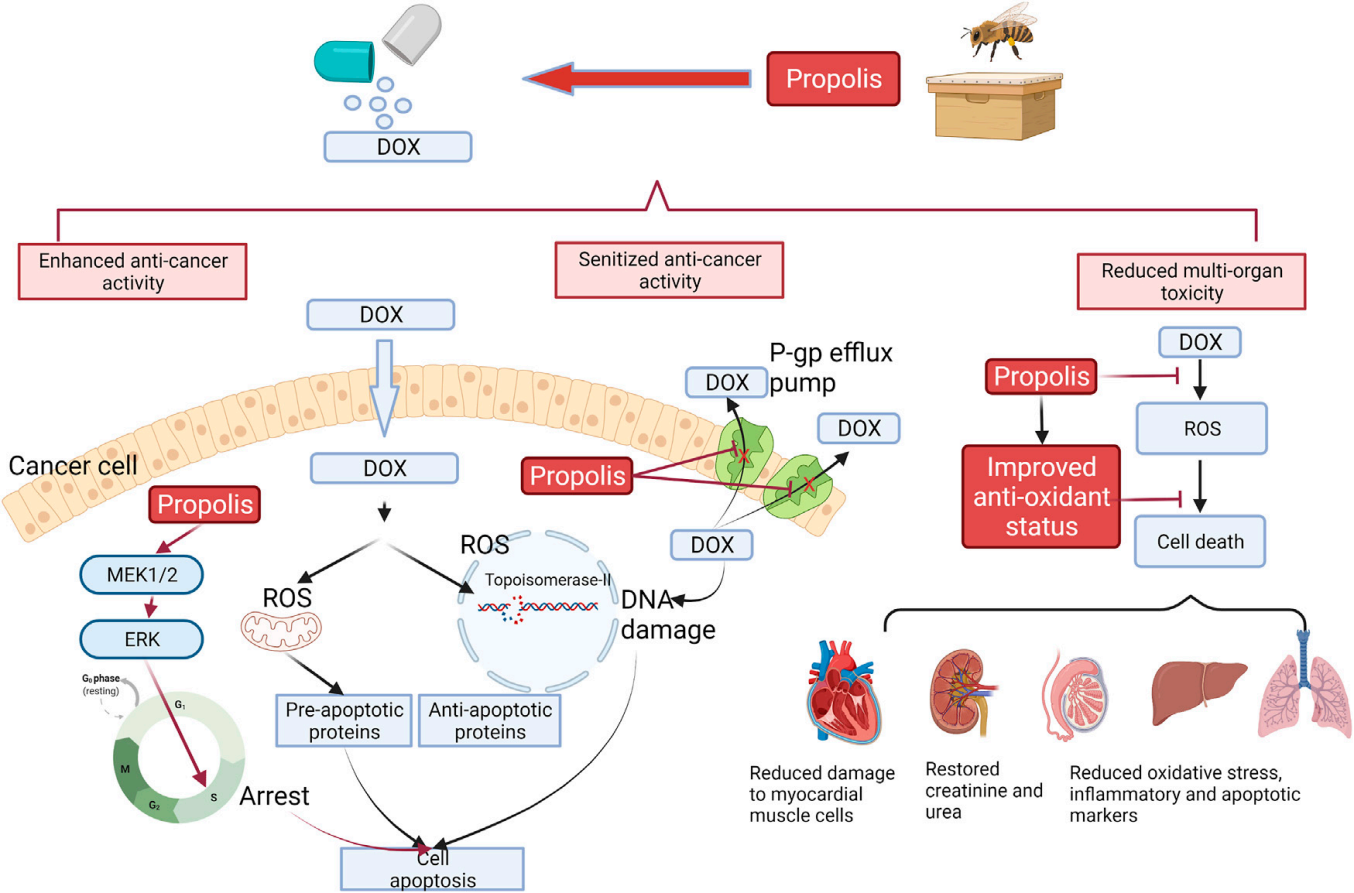
Interaction of DOX with propolis extract, which led to enhanced cell apoptosis, sensitized anti-cancer activity, and reduced multi-organ toxicity based on preclinical evidence. Black arrows represent the action of DOX, whereas red arrows represent the action of propolis. In the cancer cells, propolis was reported to induce the MEK1/2-ERK-mediated apoptotic pathway, which caused the cell cycle arrest in the S phase and strengthened the induced cell death from DOX. Propolis also inhibited the P-gp efflux pump, which increased the intracellular concentration of DOX. This action reduced the chemoresistance of cancer cells to DOX. On the contrary, the improved anti-oxidant status from propolis by scavenging ROS and increased production of anti-oxidant enzymes protected multi-organs in the body against the toxicity from DOX.

Taken together, comprehensive preclinical evidence supported the use of propolis extract to synergistically enhance and sensitize the anti-cancer activity of DOX through multiple signaling pathways on apoptosis and anti-oxidant profile and decrease the DOX-mediated side effects on multiple organs. A diagram illustrating the molecular mechanism of propolis and enhancing the efficacy of doxorubicin is shown in Figure 1.

#### Interaction With TMZ

TMZ is chemotherapy also known as an alkylating agent. It has also been widely used to treat high proliferating brain tumor cell glioblastoma multiforme (GBM) and astrocytoma attributed to its ability to cross the blood–brain barrier. TMZ is a prodrug that requires nonenzymatic hydrolysis to deliver a methylating agent to the guanine base of DNA, leading to DNA damage and triggering the death of tumor cells (Zhang, Stevens, 2012).

**FIGURE 2:**
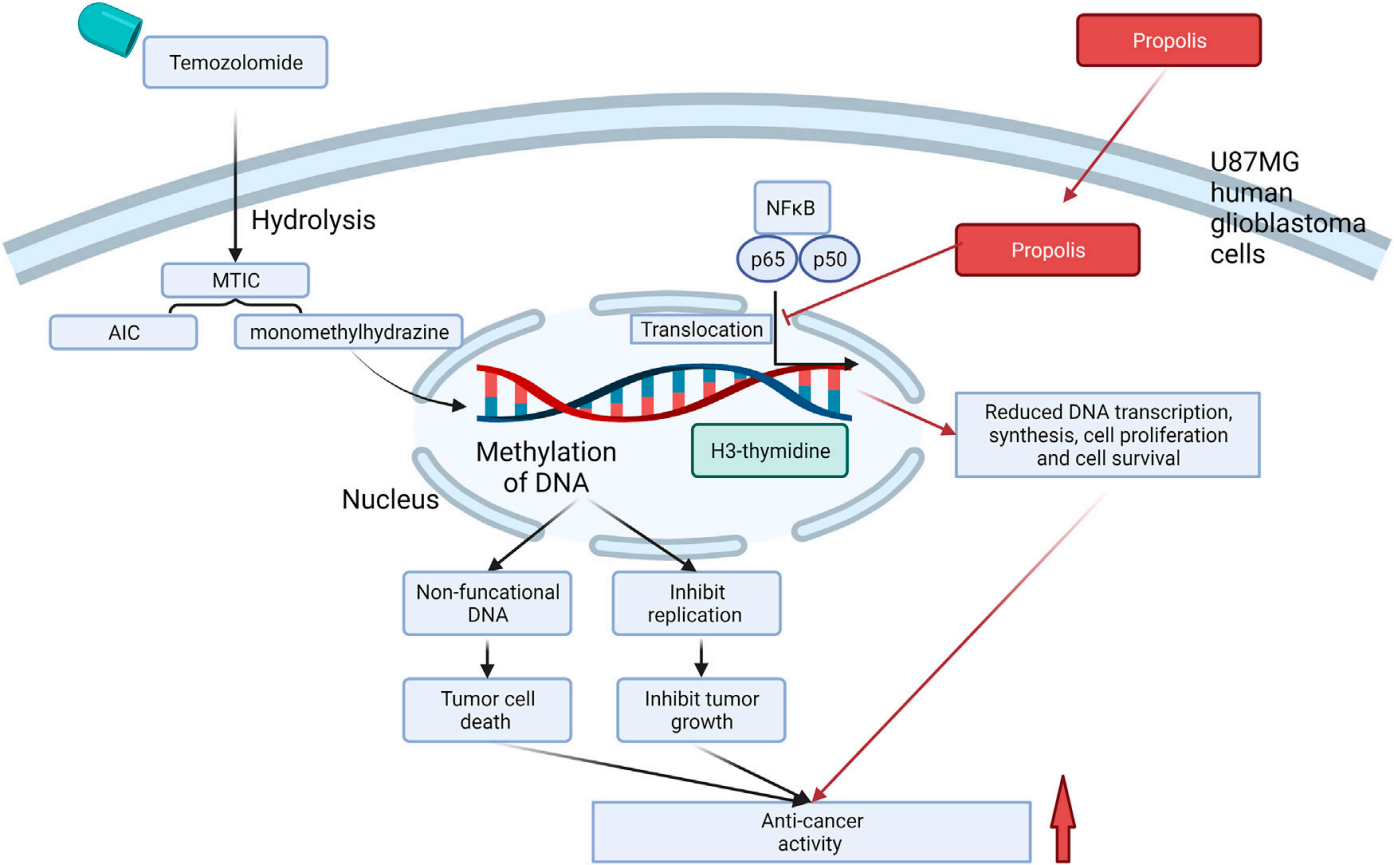
Interactions of TMZ with propolis at the molecular level, which led to enhanced cell death, in U87 MG human glioblastoma cells. Red arrow represents the molecular actions of propolis, and black arrow represents the molecular actions of TMZ. The action of TMZ that caused cell death was attributed to its metabolite-induced methylation of DNA, whereas propolis may strengthen the anti-cancer activity *via* inhibiting NF-κB signaling mediated cell proliferation and survival. MTIC and AIC are the metabolites of TMZ after hydrolysis. MIC, 3-methyl-(triazen-1-yl) imidazole-4-carboxamide; AIC, 5-aminoimidazole-4-carboxamide.

An *in vitro* study reported that the growth inhibitory effect of TMZ (20 μM) was significantly enhanced (*p* < 0.001) by the co-incubation of ethanolic extract of propolis (10–100 μg/ml) within 72 h in the U87MG glioblastoma cell line (Markiewicz-Żukowska et al., 2013). In addition, with the incorporation of H^3^-thymidine, a radiochemical marker for cell proliferation rate, the combination showed the highest inhibition of cell proliferation (by about 50%) in the U87MG cell after the 48 h exposure compared to using TMZ (no reduction) or propolis (by 20%) alone. Their results suggested that the ability of propolis to enhance the anti-cancer effect of TMZ was acted through arresting cell division and lowering DNA synthesis. The enhanced growth inhibition was likely to be associated with the action of propolis on NF-κB signaling, which is an essential survival factor for cancer cells (Godwin, Baird, 2013; Xia, Shen, 2014). The study revealed that the combination (20 μg/ml TMZ + 30 μg/ml propolis extract) significantly reduced the nuclear expressions of NF-κB subunits p65 and p50 (by approximately 50%) in U87MG cells in contrast to insignificant effects from TMZ or propolis alone. This study provides new insight into the combined action of propolis with chemotherapies *via* the action on the NF-κB pathway. A diagram illustrating the potential mechanism of propolis in enhancing the anti-cancer activity of TMZ is shown in Figure 2.

#### Interaction With Irinotecan

Irinotecan is chemotherapy widely used to treat lung cancer, colon cancer, pancreatic cancer, breast cancer, ovarian cancer, and different types of leukemia (Kciuk, Marciniak, 2020). The anti-cancer action of irinotecan is mediated by its conversion to its active metabolite SN-38 that binds to the topoisomerase-I-DNA complex and leads to the breakdown of double-stranded DNA and arrest of DNA replication and transcription (Fujita, Kubota, 2015).

Two *in vivo* studies investigated the interaction between irinotecan and ethanolic/aqueous extracts of propolis in Ehrlich ascites tumor (EAT) bearing Swiss albino mouse model. Benkovic et al. (2007) reported that either ethanolic or aqueous extract of propolis (100 mg/kg/day) combined with irinotecan (50 mg/kg/day) significantly increased the median survival time of EAT mice compared with using irinotecan alone (59.00 or 70.00 days *vs.* 39 days, *p* < 0.005) (Benkovic, Horvat Knezevic, 2007). However, only the combination of ethanol propolis and irinotecan showed a significantly enhanced antitumor effect compared to irinotecan alone, which may be due to the variation of chemical compositions between the two extracts. Later, Lisičić et al. (2014) revealed that the total flavonoids and polyphenols were substantially higher in the ethanolic extract compared to those in the aqueous extract by HPLC analysis, which might be the key to explaining the more potent anti-cancer activity of the ethanolic extract in the combination. They also reported that combining irinotecan (50 mg/kg) with ethanolic/aqueous extract of propolis (100 mg/kg) enhanced the survival rate and reduced the percentage of tumor cells in the peritoneal cavity in EAT bearing Swiss albino mice (Lisičić et al., 2014). In addition, the study has linked the mechanism of combined treatment to the immunomodulatory effect from propolis. Their results suggested a significantly (*p* < 0.05) increased population of lymphocytes, macrophages, and neutrophils in the combined group, although the increase in those cells in the propolis extracts only group was insignificant (Lisičić et al., 2014). The immunomodulatory activity of the combination has also been explored by Oršolić et al. (2010). Their results suggested that the aqueous/ethanolic extracts of propolis and related flavonoids including naringin and quercetin significantly increased the percentage of macrophage in the peritoneal cavity, which in turn protected blood, liver, and kidney cells against the toxicity of irinotecan and thus extended the survival time (Oršolić et al., 2010).

#### Interaction With 5-FU

5-FU is chemotherapy widely used to treat colorectal cancer and solid tumors in the breast, rectum, ovary, bladder, and liver. It is an analog of uracil and can act as an anti-metabolite to inhibit DNA synthesis and prevent tumor growth (Longley, Harkin, 2003). Major limitations reported for 5-FU were chemoresistance and side effects, including cytopenia and anemia along with bleeding, loss of appetite and taste, diarrhea, and feeling sick. Cytopenia and anemia induced by 5-FU were mainly attributed to the action on hemolysis, bone marrow infiltration, and disruption of erythropoiesis (Avendaño and Menendez, 2015; Bryer and Henry, 2018).

Two studies demonstrated the potential capability of propolis to enhance the anti-cancer activity and reduce the toxicity of 5-FU *via* the immunomodulatory and anti-inflammatory pathways. Suzuki et al. (2002) investigated the oral administration of crude water-soluble extract of propolis with 5-FU (50 mg/kg/day, subcutaneously) in EAT bearing mouse model (Suzuki et al., 2002). Their results demonstrated that the co-administration significantly inhibited tumor growth compared to using 5-FU alone. In addition, they noticed that the peritoneal injection of propolis into neonatal mice resulted in an increased lymphocyte/polymorphonuclear leukocyte ratio activity, indicating that the enhanced anti-cancer activity may be attributed to the capability of propolis in stimulating multicellular immunity. In addition, Sameni et al. (2021) suggested a further reduced number of aberrant crypt foci and pathological lesions in the co-administration group of ethanolic propolis extract and 5-FU in comparison to the cancer control and 5-FU monotreatment group (*p* < 0.05) in the colorectal cancer mice. Their study has linked the enhanced anti-cancer activity in the combination to the observed anti-inflammatory activity by reducing the expression of COX-2, iNOS, and β-catenin proteins (Sameni, Yosefi, 2021).

In contrast, propolis appeared to ameliorate the side effects of 5-FU on cytopenia and anemia. Suzuki et al. (2002) showed that the co-administration of propolis and 5-FU in the EAT bearing mice restored the white and red blood cell counts compared to 5-FU alone (*p* < 0.05), although no effect was observed on the platelet counts (Suzuki, Ikukatsu, Hayashi, Ikuo, 2002).

#### Interaction With MMC

MMC is an antitumor antibiotic that can inhibit DNA synthesis by cross-linking adenine at the N6 position and guanine at O6 and N2 positions. A reduced form of MMC can also cause a single-strand break in DNA (Anderson, Berberovic, 2012). Similar to 5-FU, MMC is widely used in the treatment of adenocarcinomas of the colon, breast, bladder, pancreas, and esophagus, but the efficacy is limited due to its bone marrow toxicity and induced cytopenia and anemia (Becouarn, Brunet, 1988).

A number of preclinical studies suggested that the co-administration of propolis with MMC resulted in increased tumor regression and reduced bone marrow toxicity, in which the protective mechanism may be related to the immunomodulatory and antioxidant activities by propolis. An *in vitro* investigation showed that the individual treatment of Turkish propolis and MMC exhibited significant effects in reducing cell division in human transitional carcinoma cells (Erhan Eroğlu, Özkul, 2008). When used together, the ethanolic solution of propolis was found to restore cell viability and reduce the apoptotic cell population in MMC-induced cytotoxicity in leucocytes (Al-Halbosiy, 2008). In EAT mice, the co-administration of the aqueous extract of propolis (13 mg/kg/day, oral) and MMC (1 mg/kg/day, subcutaneous) showed an enhanced antitumor effect compared to the monotherapy with MMC within 2–5 weeks, although the effect of propolis alone in tumor growth was not investigated. In addition, the WBC and RBC count increased significantly (*p <* 0.01) in the combined group compared to that of MMC alone, especially at the later stage of the chemotherapeutic course (Suzuki et al., 2002). The co-administration of Indian propolis and MMC also resulted in a significant recovery against the geno- and cytotoxic effects of MMC in bone marrow in Swiss albino mice (Kumari, Naik, 2016) and ameliorated testicular toxicity in adult male mice (Kumari, Nayak, 2017). Both studies have linked the protective effect of propolis to its substantial free radical scavenging activities, in which propolis was observed to decrease oxidative stress, reduce DNA damage, and restore tissue function (Kumari et al., 2017). Thus, the results together supported the benefits of propolis as an adjuvant therapy to promote the anti-cancer activity of MMC, as well as reducing MMC-induced cytopenia and related organ toxicity.

#### Interaction With PDT

PDT is a modern phototherapeutic approach that creates a photochemical reaction under a certain wavelength and generates ROS to selectively kill pathogens in a local area by damaging the cellular components and blood vessels that supply nutrition (Zhou Z et al., 2016). PDT has a wide range of medical applications such as skin cancer, fungal infection, tissue repair, and healing. Because of the localized action and selective uptake in the cancer cells, PDT exhibits adverse effects on normal tissues lower than chemotherapies (dos Santos, de Almeida, 2019). However, PDT also exhibits phototoxicity to the skin that causes swelling, pain, and inflammation, which is considered its major drawback (Gollnick, Evans, 2003). Thus, it is often used together with chemotherapy to reduce the dose leading to lower side effects.

**FIGURE 3:**
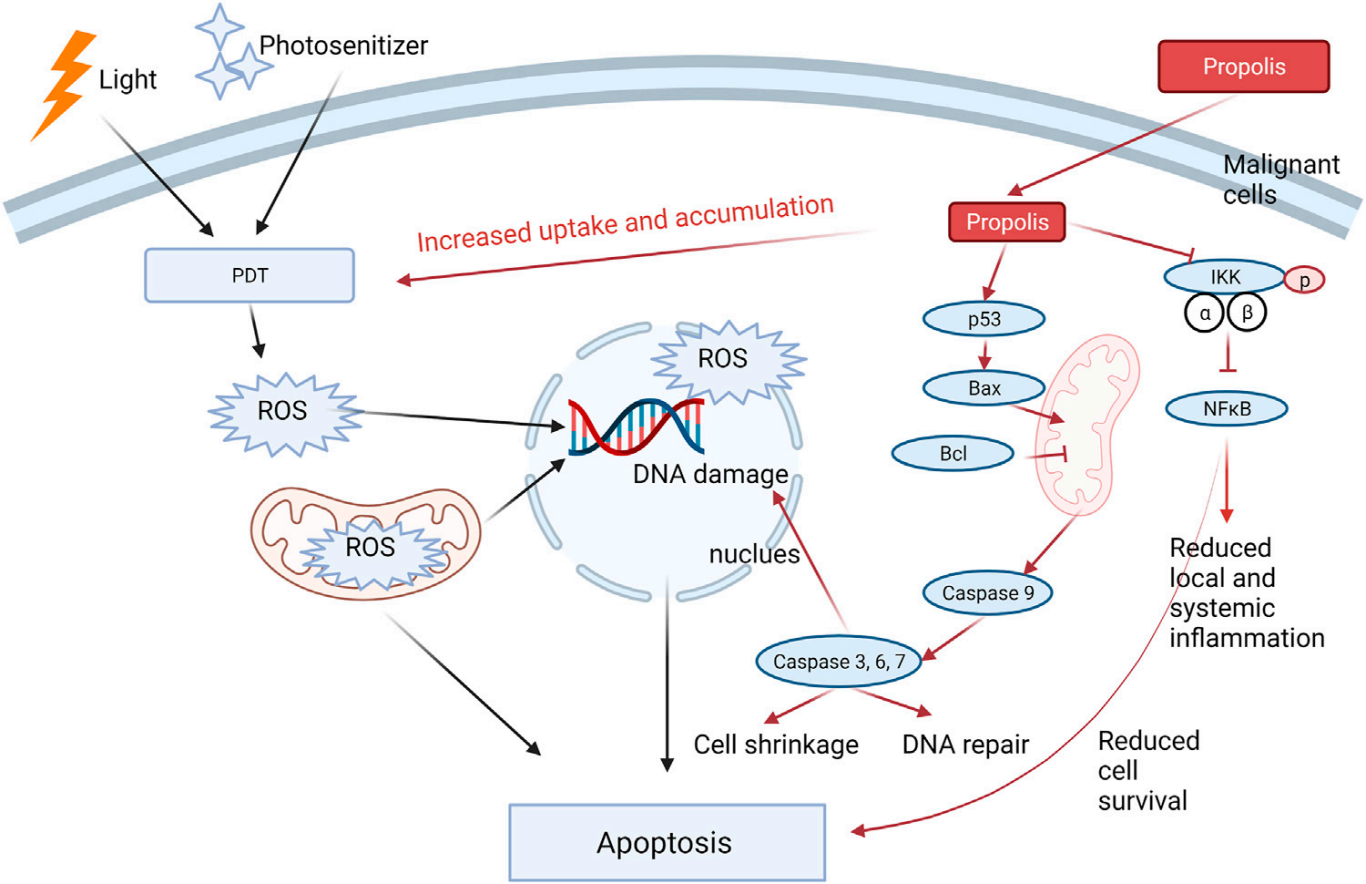
Propolis increased the cytotoxic effect of PDT through three possible mechanistic actions: 1) increased the intracellular uptake and accumulation of PDT; 2) enhanced the apoptotic signaling by regulating the pro-apoptotic and apoptotic proteins; 3) Inhibited pIKK-NFκB signaling, leading to reduced cell survival. In addition, propolis may reduce the local and systemic inflammation through the inhibition of the NF-κB pathway. Red arrows represent the action of propolis in the combination, whereas black arrows represent the action of PDT.

Two *in vitro* studies demonstrated the enhanced cytotoxic effect by combining propolis and PDT in epidermoid carcinoma cell line A431 (Ahn et al., 2013) and human head and neck cancer cells AMC-HN-4 cells (Wang et al., 2017). Both studies showed greater inhibitions of cell viability and increased apoptotic level by the combination (Ahn et al., 2013) suggested that the combination further upregulated the apoptotic proteins, including caspase-3, caspase-8, caspase-9, and poly(ADP-ribose) polymerase, which may contribute to the observed enhancement in the cytotoxic effect. Wang et al. (2017) further confirmed the synergistic cytotoxic activity of the combination in A431 cells with statistical analysis using the CI model. They also showed that the increased induction of apoptosis in the combination was related to the regulation of the pro-apoptotic proteins (Bax, NOXA, and cleaved caspase-3) and antiapoptotic protein (BcL-xL). The mechanisms were related to the promoted intracellular uptake and accumulation of PDT and downregulated NF-κB pathway, which impaired the survival of the cancer cells with the co-existence of propolis. In an *in vivo* tumorigenicity assessment using the Xenograft model, their results showed that the tumor volume range and tumor weight were the lowest in the combination group compared to every single group. A diagram that illustrates the possible mechanism of propolis in increasing the antitumor effect of PDT is shown in Figure 3.

A list of the interactions between propolis and chemotherapies and their associated mechanistic actions are shown in Table 1.

**TABLE 1:** Interaction of propolis extracts with chemotherapeutic drugs and their associated mechanisms.

Chemotherapies	Propolis extracts	Source	Study type	Subjects	Key results	Molecular mechanisms	References
DOX	Ethanolic extract	Algeria	*In vitro*	Breast cancer cells (MDA-MB-231)	Potentiated antitumor effects	Inducing cell cycle arrest in the S phase	Rouibah et al. (2021)
				Normal cells (MRC-5)	Reduced multidrug resistance	Enhanced caspase-dependent apoptosis	
					Reduced cytotoxic effect in normal cells	A significant increase in intracellular DOX content by inhibiting p-gp	
	Ethanolic and methanolic extract	Algeria	*In vivo*	DOX-induced mitochondrial stress in rats	Propolis protected heart and liver tissues from oxidative stress	Mitochondria protection by reducing malondialdehyde, restoring glutathione contents and catalase and superoxide dismutase activities	Badr et al. (2015)
	Ethanolic extract	Australia	*In vitro*	MCF7 breast adenocarcinoma	Strong synergistic interaction (CI = 0.11) in inhibiting cell proliferation	Upregulated expression of pro-apoptotic protein cyclin-dependent kinase inhibitor 1B (p27), antioxidant PON2, Claspin and catalase, and downregulated expression of anti-apoptotic protein including XIAP, HSP60, and HIF-1α	Alsherbiny et al. (2021)
	Propolis capsules	Australia	*In vivo*	DOX-induced multi-organ toxicity in rats	Pre-treatment with propolis significantly ameliorated DOX-induced cardiomyopathy, hepatotoxicity, nephritis, and pneumonia	Reduced apoptosis, oxidative stress and pro-inflammatory cytokines	Mohamed et al. (2021)
	Hydroalcoholic extract	Brazil	*In vivo*	Wistar rats	The co-administration reduced chromosome damage induced by DOX compared to the group treated only with DOX	Free radical scavenging activity by the phenolic compounds in propolis	Tavares et al. (2007)
	Methanolic extracts	Cuba	*In vitro*	Human colon carcinoma cells (LoVo Dox)	Synergistic antiproliferative and cytotoxic effect	Induced cell cycle arrest	Frión-Herrera et al. (2019)
						Increased level of apoptosis	
						Marked ROS production and drastic alteration of ΔΨm	
	Ethanolic extract	Chandigarh, India	*In vivo*	DOX-induced hepatotoxicity in male rats	Administration of animals with propolis prior to DOX led to significantly reduced hepatotoxicity parameters in blood when compared to the doxorubicin-treated group.	Modulation of the oxidative damage related parameters in liver	Singla et al. (2014)
	Propolis extract	Egypt	*In vivo*	DOX-induced cardiotoxicity and nephrotoxicity in rats	Propolis exhibited protective effects against DOX-induced cardiotoxicity and nephrotoxicity	Propolis attenuated cardiac oxidation and lowered lipid level	Ali et al. (2020)
	Ethanolic extract	Egypt	*In vivo*	DOX-induced testicular toxicity in rats	Propolis extract ameliorated DOX-induced toxicity in testis without reducing its anti-cancer potential	Restored levels of testosterone, follicle-stimulating hormone (FSH), and luteinizing hormone (LH) in normal and DOX-treated rats; restored testicular activities by regulating 3b-hydroxysteroid dehydrogenase (3b-HSD) and 17b-hydroxysteroid dehydrogenase (17b-HSD); free radicals scavenging and improving antioxidant enzymes in various tissues; reduced inflammatory and apoptotic responses	Rizk et al. (2014)
	Aqueous extract	Egypt	*In vivo*	N-methyl-N-nitrosourea (MNU) induced adenocarcinoma in rats	The combination protected liver and kidney against the toxicity of DOX	Restored liver enzyme levels including albumin, globulin, ALT, AST, ALP; improved kidney function; improved activities of antioxidant enzymes	Badr et al. (2015)
	Water extract of propolis	NA	*In vivo*	DOX-induced somatic mutation and recombination in *Drosophila melanogaster*	The combined treatment led to a reduction in the frequency of recombination compared to the treatment with DOX alone	NA	Valadares et al. (2008)
	Ethanolic extract	NA	*In vivo*	DOX-induced hepatotoxicity in rats	Improved hepatoprotective effect shown as number of vacuolated hepatocytes with mild congestion in central veins	NA	Omar et al. (2016)
TEM	Ethanolic extract	NA	*In vitro*	U87MG glioblastoma	The combination therapy significantly reduced cell viability and proliferation	Reduced DNA synthesis, enhanced cell permeability, and significantly reduced NF-κB translocation	Markiewicz-Żukowska et al. (2013)
Irinotecan	Ethanolic/aqueous extracts of propolis	NA	*In vivo*	Swiss albino mice injected with EAT	The combination with the ethanolic extract of propolis increased the life span of the tumor-bearing mice and decreased proliferation of the EAT compared to using irinotecan alone	NA	Benkovic et al. (2007); Lisičić et al. (2014)
	Ethanolic/aqueous extracts of propolis	NA	*In vivo*	Swiss albino mice injected with EAT	Combined treatment with aqueous or ethanolic extracts of propolis showed enhanced antitumor activity and prolonged survival in EAT-bearing mice	NA	Lisičić et al. (2014)
	Water-soluble derivative of propolis	NA	*In vivo*	Swiss albino mice injected with EAT	The combination treatment resulted in substantial inhibitions of the growth of EAT cells; decreased genotoxic and cytotoxic to normal cells induced by irinotecan	Immunomodulatory effect regulating lymphocyte/polymorphonuclear leukocyte ratio	Oršolić et al. (2010)
5-Fu	Aqueous extract	Brazil	*In vivo*	EAT mouse model	The co-administration significantly increased tumor regression compared with using 5-Fu alone and significantly ameliorated the cytopenia induced by 5-FU	Restored white and red blood cell counts	Suzuki et al. (2002)
	Alcoholic extract	Iran	*In vivo*	AOM/DSS induced colorectal cancer in BALB-c mice	Propolis increased the anti-cancer of 5-Fu by further inhibiting the onset and progression of colorectal cancer	A greater decrease in Cox-2 and iNOS expression leading to reduced cell survival	Sameni et al. (2021)
MMC	Ethanolic extract	Turkey	*In vitro*	Human peripheral lymphocyte viability	The co-incubation of either propolis extract and MMC enhanced the cell viability of lymphocyte compared to using MMC alone	NA	Arslan et al. (2021)
	Ethanolic extract	Iraq	*In vivo*	Albino male mice	Propolis may have the potential to inhibit the genotoxic effects of MMC without compromising the anti-cancer activity of MMC	Immunomodulatory capacity of propolis through a significantly increased total count of leucocytes and mitotic index	Al-Halbosiy (2008)
	Hydroethanolic extract	India	*In vivo*	Healthy adult male mice	The co-administration protected testis against the toxicity from MMC	Reduced DNA damage, elevated the anti-oxidant activity, restored the testicular testosterone and inhibin B level	Kumari et al. (2017)
	Hydroethanolic extract	NA	*In vivo*	MMC-induced bone marrow toxicity in Swiss albino mice	Hydroethanolic extract of propolis possessed substantial geno- and cytoprotective properties against MMC	Free radical scavenging activity of propolis	Kumari et al. (2016)
	Aqueous extract	NA	*In vivo*	EAT mouse model	Significantly increased tumor regression compared to using MMC alone and attenuated cytopenia induced by MMC	Restored white blood cells, red blood cells, and platelet counts	Suzuki et al. (2002)
PDT	Ethanolic extract	Seoul, South Korea	*In vitro*	Human head and neck cancer cells AMC-NH-4	The combined treatment enhanced the inhibition of tumor cell viability and increased apoptotic level	Upregulated caspase-mediated cell apoptosis	Ahn et al. (2013)
	Ethanolic extract of green propolis	Brazil	*In vitro* and *in vivo*	Human epidermoid carcinoma A431 cell and cervical cancer HeLa cell, xenograft mouse model	Synergistic effect (CI < 1) in reducing tumor cell viability in the combination and suppressed inflammatory response	Increased PDT intracellular uptake and accumulation; upregulated Bax/Bcl-xL and caspase-mediated cell apoptotic level; inhibited pIKK-NFκB signaling pathway	Wang et al. (2017)

NA, not available.

### Interaction With Anti-Microbial Drugs

Propolis has been used against infectious diseases from ancient Greek, Roman, and Egyptian ages to modern times, and its diverse antimicrobial potential has been confirmed in numerous scientific studies (Kuropatnicki, Szliszka, 2013; Almuhayawi, 2020). Propolis and its derivatives contain a broad range of natural compounds, including polyphenols, flavonoids, and fatty acids, which have shown significant effectiveness against different types of microorganisms (Afrouzan, Tahghighi, 2018). Thus, the evaluation of antimicrobial properties of the propolis from different sources in single and combination therapy with standard antibiotics against a broad range of organisms has gained increasing focus by the researchers (Almuhayawi, 2020). Many studies have shown an enhanced or synergistic effect by combining propolis with many standard antimicrobial drugs to improve activity against the resistant microorganism (Al-Waili, Al-Ghamdi, 2012).

#### Interaction With Antibiotics

Propolis exhibited a strong and multi-targeted anti-bacterial activity mainly attributed to its flavanols components (Gonsales, Orsi, 2006). Its mechanistic actions against bacteria include the inhibition of cell division and synthesis of the cell wall, reduction of ATP production, decreasing bacterial mobility, disturbance of the membrane potential, and inducing the immune system (Almuhayawi, 2020; Przybyłek and Karpiński, 2019, Tomasz M, 2019). The antimicrobial characteristics of propolis are extremely important for the food industry attributed to its potential to increase the shelf life of food products. In addition, the multi-target function against bacteria of propolis encouraged many studies looking into the combined use of propolis to help overcome the resistance to antibiotics. The synergistic anti-bacteria activity for the combined use of propolis and antibiotics were reflected as a directly enhanced anti-bacterial effect, reduced antibiotic resistance, and organ protective effect in the body.

Despite the various extraction methods and sources of propolis, a number of microbiological studies have shown the enhanced combinatory effect of propolis extracts with different classes of antibiotics in both gram-positive and gram-negative bacteria*.* In particular, a confirmed synergy by fractional inhibitory concentration (FIC) values was demonstrated in propolis ethanolic extracts with ceftriaxone, ertapenem, and ofloxacin on *Escherichia coli* (Lavigne, Ranfaing, 2020), oxacillin, and vancomycin on various bacterial strains, including methicillin-resistant *Staphylococcus aureus* (MRSA) (Al-Ani, Issam, Zimmermann, Stefan, 2018), macrolides on *Streptococcus pyogenes* and *Haemophilus influenzae* (Speciale, Costanzo, 2006), and clarithromycin on *Helicobacter pylori* (Nostro, Cellini, 2006). Remarkably, these above-mentioned antibiotics can be classified into two types based on their mechanistic actions: 1) antibiotics that interrupt the bacterial cell wall (ceftriaxone, ertapenem, oxacillin, and vancomycin) and 2) antibiotics that inhibit bacterial protein synthesis by binding to the bacterial 50S ribosomal subunit (i.e., clarithromycin and macrolides). As these two actions were demonstrated in the action of propolis against bacteria, it is thought that synergy may have occurred because of the strengthened actions in these two pathways when using the propolis and antibiotics together. We also noticed that propolis shows additive or antagonistic interactions with antibiotics on gram-negative bacteria strains, such as *Salmonella typhi* and *Pseudomonas keratitis*, rather than gram-positive strains*.* Many previous studies have demonstrated that a single application of propolis was more effective against gram-positive (i.e., *Staphylococcus aureus*, methicillin-susceptible *Staphylococcus aureus*, and MRSA, *Enterococcus faecalis*) than some gram-negative bacteria (i.e., *Klebsiella pneumoniae* and *Escherichia coli*). The limited activity of propolis on certain gram-negative bacteria was suggested to be attributed to the species-specific structure of the outer membrane of which the production of hydrolytic enzymes compromises the action of active components in propolis (Przybyłek, Izabela and Karpiński, Tomasz M, 2019). Thus, the weakened anti-bacteria activity of propolis may lead to limited enhancement with antibiotics when used together. Another factor that may affect the synergistic observation is the susceptibility status of the bacteria stains to the antibiotics. Based on the study from Lavigne et al. (2020), the addition of propolis (hydroalcoholic extract of blended propolis in carob (60/40, w/w)) synergistically improved the bactericidal effect of ceftriaxone, ertapenem, and ofloxacin which were all active to a panel of empathogenic *E. coli*. However, no synergistic interaction was detected between propolis and fosfomycin on the tested strains, which were all resistant to fosfomycin (MIC values > 128 mg/L).

The beneficial use of propolis and antibiotics was also manifested as organ protective activity in the body. Two *in vivo* studies demonstrated that the co-administration of propolis and cefixime improved the overall status of *Salmonella enteric*-infected mice by reducing bacterial load, improving survival, restoring hematological parameters, and alleviating the toxicity to the kidney, spleen, and liver (Kalia, Kumar, 2016; Przybyłek, Izabela and Karpiński, Tomasz M, 2019). The organ-protective effect of propolis was linked with its strong antioxidant property as a scavenger of free radicals.

It was worth mentioning that most studies suggested the enhanced effect of the combination by comparing the zone of inhibition or minimum inhibitory concentrations (MIC) rather than determining the FIC index (synergy refers to FIC index ≤0.5). Thus, their determination on the synergistic interaction is deemed not conclusive. In addition, the observed synergistic effects were generally demonstrated in *in vitro*, and the confirmation in *in vivo* and human trials is lacking to define the real efficacy. A summary of the combined effect of antibiotics with propolis extract is shown in Table 2.

**TABLE 2:** Interaction of propolis in combination with different antibiotics.

Antibiotics	Propolis extract	Source	Test microorganisms	Interaction	Key results	References
Ampicillin	Ethanolic extract	Iraq	*Salmonella typhi*	Enhanced anti-bacterial effect	Significantly enlarged zone of inhibition using the combination compared to using ampicillin alone	AL-safi (2013)
Beta-lactams (amoxicillin, ampicillin, amoxicillin/clavulanic acid, cefixime, erythromycin)	Alcoholic (76%) or hydroglyceric extracts (30%) of propolis	NA	Respiratory infectious strains *(Streptococcus pneumoniae*, *Haemophilus influenzae*, *Haemophilus parainfluenzae*, *Moraxella catarrhalis*, and *Streptococcus pyogenes)*	Additive or antagonistic	Combinations with either propolis extract generally showed additive or antagonistic activities, as shown by FIC values	Speciale et al. (2006)
Cefoxitin	Ethanolic extract	Poland	*Staphylococcus aureus* and methicillin-resistant *Staphylococcus aureus*	Enhanced anti-bacterial effect	Stronger anti-bacterial effect shown as a larger diameter of inhibition compared to each monotherapy	Wojtyczka et al. (2013)
Cefixime	Ethanolic extract	India	*Salmonella enteric* in mice	Enhanced anti-bacterial effect; reduced toxicity	Reduced bacterial load, improved survival, restored hematological parameters, and prevented bacteria-induced toxicity to kidney, spleen, and liver	Przybyłek and Karpiński (2019)
			*Salmonella*-infected BALB/c mice	Organ protective effect	Both the combinations and cefixime were effective in reducing bacterial counts in the body after 5 days of treatment. However, propolis showed protective effects on liver, spleen, and kidney functions	Kalia et al. (2016)
Ceftriaxone	Hydroalcoholic extract of blended propolis mixed with carob in a proportion of (60/40, w/w)	NA	*Escherichia coli*	Synergistic	Propolis improved the effect of ceftriaxone and showed a synergistic bactericidal effect as evidenced by FIC value compared with using ceftriaxone alone	Lavigne et al. (2020)
Chloramphenicol	Ethanolic extract	Brazil	*Staphylococcus aureus*	Enhanced anti-bacterial effect	The combination significantly increased the zone of inhibition compared with using gentamycin alone as assessed by the Kirby and Bauer method and comparison of MIC values	Fernandes Júnior et al. (2005)
		Bulgaria	*Salmonella typhi*	No interaction	No positive interaction was found	Orsi et al. (2012)
Ciprofloxacin	Ethanolic extract	USA	*Pseudomonas keratitis* in rabbits	No interaction	Both the mean bacterial counts and corneal opacity scores in the combination were statistically the same (*p* > 0.05) compared to those of ciprofloxacin alone	Onlen et al. (2007a)
Clarithromycin	Ethanolic propolis extract	NA	Clinical strains of *Helicobacter pylori*	Synergistic or additive	The combinations exhibited an improved inhibition of *H. pylori* with synergistic or additive activity as shown by FIC values, although the MIC values were generally higher than those of clarithromycin alone	Nostro et al. (2006)
Clindamycin	Ethanolic extract	Brazil	*Staphylococcus aureus*	Enhanced anti-bacterial effect	The combination significantly increased the zone of inhibition compared with using gentamycin alone as assessed by comparison of MIC values	Fernandes Júnior et al. (2005)
	Ethanolic extract	Poland	*Staphylococcus aureus* and methicillin-resistant *Staphylococcus aureus*	Enhanced anti-bacterial effect	Stronger anti-bacteria effect shown as the larger diameter of inhibition as compared to each monotherapy	Wojtyczka et al. (2013)
Cotrimoxazol	Ethanolic extract	Brazil	*Staphylococcus aureus*	Enhanced anti-bacterial effect	The combination significantly increased the zone of inhibition compared with using gentamycin alone as assessed by the Kirby and Bauer method	Fernandes Júnior et al. (2005)
Erythromycin	Ethanolic extract	Poland	*Staphylococcus aureus* and methicillin-resistant *Staphylococcus aureus* (gram-positive)	Enhanced anti-bacterial effect	Stronger anti-bacteria effect shown as larger diameter of inhibition compared to each monotherapy	Wojtyczka et al. (2013)
Ertapenem	Hydroalcoholic extract of blended propolis mixed with carob in a proportion of (60/40, w/w).	Various origins	*E. coli (gram-negative)*	Synergistic	Propolis improved the effect of ertapenem and showed a synergistic bactericidal effect as evidenced by FIC value compared with using ceftriaxone alone	Lavigne et al. (2020)
Fluoroquinolones	Alcoholic (76%) and hydroglyceric extracts (30%) of propolis	NA	Respiratory infectious strains *(Streptococcus pneumoniae*, *Haemophilus influenzae*, *Haemophilus parainfluenzae*, *Moraxella catarrhalis*, and *Streptococcus pyogenes)*	Additive or antagonistic	Combinations with either propolis extract generally showed additive or antagonistic activities, as shown by FIC values	Speciale et al. (2006)
Gentamicin	Hydroethanolic red propolis collected from different seasons	Brazil	*Escherichia coli*, *Pseudomonas aeruginosa*, and *Staphylococcus aureus*	Enhanced anti-bacterial effect	Combination with red propolis collected in the dry season showed significantly lower MIC value compared with using gentamicin alone	Neto et al. (2017)
	Ethanolic extract	Iraq	*Salmonella typhi*	No interaction	No significant difference was shown between the combination and using gentamycin alone by comparing the zone of inhibition	AL-safi (2013)
	Ethanolic extract	Brazil	*Staphylococcus aureus*	Enhanced anti-bacterial effect	The combination significantly increased the zone of inhibition compared with using gentamycin alone as assessed by the Kirby and Bauer method and comparison of MIC values	Fernandes Júnior et al. (2005)
Imipenem	Hydroethanolic red propolis collected from different seasons	Brazil	*Pseudomonas aeruginosa* and *Staphylococcus aureus*	*Pseudomonas aeruginosa:* enhanced anti-bacterial effect	Combination with red propolis collected in the dry season showed significantly lower MIC value compared with using imipenem alone against *P. aeruginosa*; no improvement was shown against *S. aureus*	Neto et al. (2017)
				*Staphylococcus aureus:* no interaction		
Linezolid	Ethanolic extract	Poland	*Staphylococcus aureus* and methicillin-resistant *Staphylococcus aureus*	Enhanced anti-bacterial effect	Stronger anti-bacteria effect shown as larger diameter of inhibition compared to each monotherapy	Wojtyczka et al. (2013)
Levofloxacin	Ethanolic extract	Germany, Ireland, and the Czech Republic	*Streptococcus pyogenes*, *Haemophilus influenzae*, *Streptococcus pyogenes*	Synergistic	Synergistic interaction against all tested strains as assessed by MIC and FIC values	Al-Ani et al. (2018)
Macrolides	Alcoholic (76%) and hydroglyceric extracts (30%) of propolis	NA	Respiratory infectious strains *(Streptococcus pneumoniae*, *Haemophilus influenzae*, *Haemophilus parainfluenzae*, *Moraxella catarrhalis*, and *Streptococcus pyogenes)*	Additive or antagonistic	Combinations with either propolis extract generally showed additive or antagonistic activities, as shown by FIC values	Speciale et al. (2006)
Mupirocin (topical)	Ethanolic extract of propolis	NA	Methicillin-resistant *S. aureus* infected rabbits	Enhanced anti-bacterial effect	Significantly lowered bacterial count and polymorphonuclear leukocyte in nasal mucous membrane in rats compared with the combination and each respected monotherapy	Onlen et al. (2007b)
Neomycin	Ethanolic extract of propolis	Brazil or Bulgaria	*Salmonella typhi*	No interaction	No positive interaction was found	Orsi et al. (2012)
Netilmicin	Ethanolic extract	Brazil	*Staphylococcus aureus*	Enhanced anti-bacterial effect	The combination significantly increased the zone of inhibition compared with using gentamycin alone as assessed by the Kirby and Bauer method and comparison of MIC values	Fernandes Júnior et al. (2005)
Ofloxacin	Hydroalcoholic extract of blended propolis mixed with carob in a proportion of (60/40, w/w).	various origins	*E. coli*	Synergistic	Propolis improved the effect of ofloxacin and showed a synergistic bactericidal effect as evidenced by FIC value compared with using ofloxacin alone	Lavigne et al. (2020)
Oxacillin	Ethanolic propolis	Germany, Ireland, and the Czech Republic	*MRSA*	Synergistic	Synergistic interaction as assessed by MIC and FIC values	Al-Ani et al. (2018)
Penicillin	Ethanolic extract	Poland	*Staphylococcus aureus* and methicillin-resistant *Staphylococcus aureus*	Enhanced anti-bacterial effect	Stronger anti-bacterial effect shown as larger diameter of inhibition as compared each monotherapy	Wojtyczka et al. (2013)
Tetracycline	Ethanolic extract	Brazil or Bulgaria	*Salmonella typhi*	No interaction	No positive interaction was found	Orsi et al. (2012)
	Ethanolic extract	Brazil	*Staphylococcus aureus*	Enhanced anti-bacterial effect	The combination significantly increased the zone of inhibition compared with using gentamycin alone as assessed by the Kirby and Bauer method and comparison of MIC values	Fernandes Júnior et al. (2005)
	Ethanolic extract	Poland	*Staphylococcus aureus* and methicillin-resistant *Staphylococcus aureus*	Enhanced anti-bacterial effect	Stronger anti-bacterial effect shown as larger diameter of inhibition as compared each monotherapy	Wojtyczka et al. (2013)
Tobramycin	Ethanolic extract	Poland	*Staphylococcus aureus* and methicillin-resistant *Staphylococcus aureus*	Enhanced anti-bacterial effect	Stronger anti-bacterial effect shown as larger diameter of inhibition as compared each monotherapy	Wojtyczka et al. (2013)
Trimethoprim + sulfamethoxazole	Ethanolic extract	Poland	*Staphylococcus aureus* and methicillin-resistant *Staphylococcus aureus*	Enhanced anti-bacterial effect	Stronger anti-bacterial effect shown as larger diameter of inhibition as compared each monotherapy	Wojtyczka et al. (2013)
Vancomycin	Ethanolic extract	Germany, Ireland, and the Czech Republic	MRSA, *E. faecalis*, *S. pneumonia*, *S. pyogenes*, and *H. influenza*	Synergistic	Strong synergistic interaction (CI = 0.38–0.5) to inhibit the growth of gram-positive bacteria than gram-negative bacteria was reported	(Al-Ani et al., 2018)
		Brazil	*Staphylococcus aureus*	Synergistic	Kirby and Bauer and E-test methods revealed synergism	Fernandes Júnior et al. (2005)
		Germany, Ireland, and the Czech Republic	*MRSA*, *E. faecalis*, *S. pyogenes*	Synergistic	Synergistic interaction against all tested strains as assessed by MIC and FIC values	Al-Ani et al. (2018)

#### Interaction With Antifungal Drugs

Growing resistance to antifungal drugs and re-occurrence of fungal infections are the two major challenges for antifungal therapies due to the eukaryotic nature of the fungus. Thus, powerful action of antifungal therapy to completely eradicate the organism is desired (Metin, Dilek, 2018). However, limited therapeutic options and inappropriate use of antifungal drugs cause the selection of resistant micro-organisms. Resistance to antifungal therapies can be developed *via* altered drug permeability, modification of the target site, formation of biofilms, and reduced intracellular drug level by efflux pump (Cowen, Sanglard, 2014). In recent years, the antifungal activity of propolis has been reported against a wide variety of fungi (Siqueira, Gomes, 2009; Dalben-Dota, Faria, 2010). With the growing incidence of antifungal resistance, especially with the *Candida* spp., combinations of propolis extract with antifungal drugs including fluconazole, anidulafungin, and nystatin were investigated.

Stepanović et al. (2003) suggested a synergistic effect of combining ethanolic extract of propolis and nystatin (100 IU) against *C. albicans* compared to propolis extract alone (Stepanović et al., 2003) as determined by the disc diffusion method. Pippi et al. (2015) showed a synergistic interaction (FICI ≤5) between n-hexane extract of Brazilian red propolis and fluconazole combination against five resistant clinical isolates of *C. parapsilosis* and *C. tropicalis* manifested by a significantly impaired survival (*p <* 0.05) compared to the single therapy of fluconazole. However, no synergism was observed for the combination of propolis extract with anidulafungin against the tested fungal species compared to anidulafungin alone, although obvious cell damage was detected (Pippi, Lana, 2015). Two studies showed that the antifungal activity of propolis was associated with inhibiting the synthesis of the fungal cell wall formation and biofilm *via* inhibiting the formation of β-1, 3-D-glucan (Flevari, Theodorakopoulou, 2013; Leite, Martins, 2020). Thus, it is speculated that synergistic interaction between propolis and fluconazole was likely attributed to the action of propolis to damage the fungal cell wall, facilitating intracellular transportation of fluconazole with high permeability. On the contrary, the absence of synergy between propolis and anidulafungin was likely due to the similar model of action on the cell wall and thus no interference. Although further investigation is warranted to confirm the mechanism, these findings have shed light on future research, searching for a synergistic combination of propolis and antifungal medications to a more powerful therapeutic outcome and reduced resistance.

### Interaction With Metformin

Type II diabetes (T2D) is a metabolic disorder characterized as reduced secretion of insulin or insulin resistance resulting in persistent hyperglycemia (Asmat, Abad, 2016). The pathogenesis of T2D includes oxidative and inflammatory damage of the pancreatic β cells and the altered expression of regulatory genes (Cernea and Dobreanu, 2013). Metformin is a first-line T2D therapy that effectively reduces glucose production and increases sensitivity to glucose by modulating lipid metabolism and enhancing peripheral glucose uptake and utilization. Nevertheless, the efficacy of metformin on T2D related complications such as organ damage is limited to its mono-action (Nasri and Rafieian-Kopaei, 2014; Pernicova and Korbonits, 2014). Propolis has been found to protect pancreatic cells from oxidative damage induced by free radicals, contributing to the restoration of the normal production of insulin from the pancreas and thus significantly lowering blood glucose level (Kitamura, 2019). The anti-oxidant and anti-inflammatory properties of propolis were suggested to be beneficial for protecting the reproductive system (Nna et al., 2019, Nna et al., 2021; Nna et al., 2020) and kidney function (da Costa, Libório, 2015; Silveira, Teles, 2019) against T2D. Thus, it is plausible that propolis combined with the metformin offers an enhanced therapeutic outcome in treating T2D and related complications.

Nna et al. (2018b) combined the ethanolic extract of Malaysian propolis (300 mg/kg b.w.) with metformin (300 mg/kg b.w.) on STZ-induced diabetic rats, and their results demonstrated a significant reduction of glucose level by both metformin (*p* < 0.01) and propolis monotherapy (*p* < 0.01). Noticeably, the combination therapy showed the lowest blood glucose level, a 1.69-fold decrease compared to metformin alone (1.43-fold). The enhanced anti-hyperglycemic activity in the combination may be due to the direct action of propolis as insulin mimetics to increase the usage or sensitivity toward glucose (Yeh, Eisenberg, 2003) and/or inhibition of α-glucosidase (Ibrahim et al., 2016). In addition, the degeneration of pancreatic islets as a result of oxidative stress negatively affects circulating insulin level and results in persistent hyperglycemia. Thus, the anti-oxidant, anti-inflammatory, and anti-apoptotic actions of propolis were thought to enhance the anti-glycemic activity indirectly by a regenerative effect on pancreatic β-cells (Nna et al., 2018a).

**FIGURE 4:**
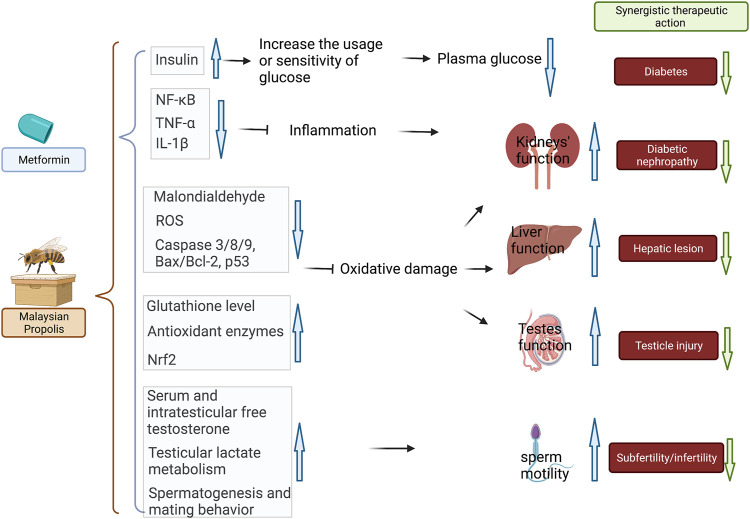
Combined therapy of Malaysian propolis and metformin achieved the most prominent results in treating diabetes, diabetic nephropathy, attenuating hepatic, testicle injury, and subfertility/infertility in diabetic mice in comparison to the mono-therapeutic interventions. The possible mechanistic actions in attenuating hyperglycemia and diabetes-related renal, hepatic, and testicle damage were related to 1) increased insulin sensitivity and glucose uptake; 2) reduced inflammation *via* NF-κB pathway; 3) antioxidant and reduced oxidative damage *via* activating Nrf2-regulated antioxidant genes. The therapeutic benefits for subfertility and infertility were related to regulated serum, intratesticular free testosterone, testicular lactate metabolism, and spermatogenesis and mating behavior. Black arrows represent the action of metformin in the combination, whereas brown arrows represent the action of propolis.

A series of studies demonstrated the synergistically combined activities of Malaysian propolis and metformin in mitigating oxidative stress and inflammation in the kidney, liver, testes, and reproductive system in diabetic mice/rats (Nna et al., 2019; Nna et al., 2021; Nna et al., 2020; Nna et al., 2018b). All of these studies confirmed the most activity by propolis and metformin combination in lowering the FBG level in STZ-induced mice compared with using propolis or metformin alone. In addition, the combined group also showed the most potent activity in restoring renal, liver, and testes functions compared with each monotherapy. The prominent renal, liver, and testes protective activities were associated with the action of propolis in reducing oxidative stress (inducing anti-oxidant enzymes), inhibiting inflammatory markers (NF-κB, TNF-α, IL-1β, and IL-10), and decreasing apoptotic proteins (Bax/Bcl-2, p53, caspase-8, caspase-9, and caspase-3). In particular, propolis and metformin mitigated subfertility in STZ-induced diabetic male rats associated with the action of propolis in upregulating testicular monocarboxylate transporter (MCT) 2, MCT4, and lactate dehydrogenase type C mRNA levels and improving sperm parameters and sperm nDNA fragmentation. Interestingly, the single action of propolis was comparable to that of metformin, but better effects were generally observed in organ protection when the co-administration was used (Nna et al., 2020). The interaction of Malaysian propolis and metformin is summarized in Figure 4.

### Interaction With Praziquantel

Schistosomiasis is a devastating parasitic disease caused by *Schistosoma mansoni*, which is mainly spread by freshwater snails and can infect humans through the skin. It can cause an inflammatory reaction and progressive organ damage if left untreated for a long period of time. Praziquantel is the only available anti-parasitic drug for treating schistosomiasis (MacConnachie, 2012; Vale, Gouveia, 2017). Praziquantel increases the intracellular Ca^2+^ influx to enhance the muscle contraction of *S. mansoni*, which in turn induces severe spasms and paralysis of muscles to cause worm contraction and death (Xiao, Sun, 2018). However, decreased sensitivity of praziquantel was reported in mature parasites, which often requires the repetition of the therapy in chronic infection to prevent recurrence (King, Olbrych, 2011; Coeli, Baba, 2013; Bergquist, Utzinger, 2017). The effect of propolis against human parasites such as protozoa, helminths, malaria has been reported by several studies (Siheri, Ebiloma, 2019; Paula et al., 2021; Rivera-Yañez, Rivera-Yañez, 2021). Interestingly, the oral treatment of Brazilian red propolis (25 μg/ml) was effective against adult schistosomes (100% mortality) as evidenced by morphological alterations in the tegument of schistosomes, and it was more effective against adult schistosomes (chronic infection) than the immature stage (early infection) (Silva, Silva, 2021). Thus, the combinatory therapy of propolis and praziquantel may represent a plausible therapy as an advanced and long-lasting anti-parasitic efficacy (Dantas Silva, Machado, 2017; de, Cândido, 2021).

The major target for the anti-schistosomal agent is the tegument of *Schistosoma* to impair the parasite’s survival and/or the host immune defense. When used together, propolis was shown to enhance the effectiveness of praziquantel related to its action through the host immune defense. Propolis also exhibited hepatoprotective activity against the damage from the infection. Mahmoud et al. (2014) investigated the combination of ethanolic extract of Egyptian propolis (300 mg/kg) and praziquantel (500 mg/kg) in *Schistosoma mansoni-*infected Swiss albino mouse model. The combination therapy showed the most effective action (*p* < 0.01) in reducing worm burden compared to the infected model control and each monotherapy, although propolis extract alone showed a slight reduction of worm burden. In addition, the combination showed a pronounced hepatoprotective activity manifested as significantly alleviated inflammation and fibrosis. The improved biochemical parameters included plasma proteins, reduced histological parameters of infection, and improved immunological parameters such as IgG and IgM antibodies (*p* < 0.01 *vs.* praziquantel mono-therapy). A protective effect against *S. mansoni*-induced damage was enhanced by the combination therapy with a reduction in the degree of lymphocytic infiltration (*p <* 0.05), aggregation (*p <* 0.05), hepatic granulomatous lesions (*p <* 0.01), and lipid peroxidation (*p <* 0.05) compared to the single praziquantel therapy, indicating the reduction of *S. mansoni* infection-induced inflammation through the immunomodulatory action (Rizk et al., 2014). Similarly, *S. mansoni* infected mice treated with a combination of praziquantel (500 mg/kg/d for 2 days beginning 4 weeks after infection) and propolis (250 mg/kg/d during 5th to 12th weeks after infection) resulted in a significant reduction in hepatic hydroxyproline build-up/liver pathologies, which were comparable or more potent than each monotherapy through the immune-modulatory effects on immunoglobulin E (IgE), IgG, alanine transaminase (ALT), aspartate aminotransferase (AST), and hepatic hydroxyproline levels (El-Sisi, Awara, 2011).

### Interaction With Donepezil

As the most common cause of dementia, Alzheimer’s disease is characterized by progressive and irreversible cognitive and memory loss, affecting an increasing population of the elderly worldwide (Pisani, Mueller, 2021). Remarkably, recent studies have shown that people with mild cognitive impairment, although is normal in aging, have a three to five times higher risk of developing dementia, especially Alzheimer’s disease (Bennett, Wilson, 2002). Donepezil is in the medications class of cholinesterase inhibitors, which is indicated for mild to moderate Alzheimer’s disease to attenuate the clinical symptoms (Grossberg, 2003). The efficacy of donepezil in improving memory relies on its specific action of increasing cholinergic transmission that plays an important role in short-term memory. Thus, donepezil has also been discussed in the position to improve memory for healthy older individuals to prevent or reduce the risk of Alzheimer’s disease (Schredl, Weber, 2001; Beglinger, Tangphao-Daniels, 2005; FitzGerald, Crucian, 2008). On the contrary, many studies have shown promising neuroprotective and anti-neuroinflammatory properties of propolis (Nakajima et al., 2007; Nakajima et al., 2009; Li, Chu, 2019), and thus it has been considered as a useful adjuvant therapy in neurological disorders such as Alzheimer’s disease (Zulhendri, Perera, 2021).

Ayikobua et al. (2018) investigated the combined effect of donepezil and ethanolic extract of propolis (source not specified) in wild-type *Drosophila melanogaster* (*n* = 10 each group) (Ayikobua, Semuyaba, 2018). Their results demonstrated that 10 ml of donepezil (0.001 M) combined with 50 mg propolis in food exhibited a time-dependent improvement trend in the short- and long-term memory for 30 days. In particular, the improvement in the long-term memory in the combined group appeared to be markedly higher than that of the single donepezil (0.001 M) or single propolis (50 or 250 mg/ml) groups at the end of the intervention. However, it was uncertain whether the short-term and long-term memory of all the flies were comparable at the baseline, and there was a lack of statistical analysis on the comparison among groups, which may lead to a biased conclusion.

### Interaction With Levodopa

Parkinson’s disease (PD) describes the abnormality of movement caused by a disorder of the central nervous system (Poewe, Seppi, 2017). It has gained increasing popularity among the elderly worldwide, partially attributed to the longer disease duration and environmental factors (Houston and McGill, 2013). Levodopa is the first-line medication for PD, which acts as a non-competitive antagonist to boost dopamine release and prevent dopamine reuptake. However, the long-term use of levodopa is also associated with a series of adverse reactions and loss of efficacy. Since the increasing dose of levodopa likely leads to higher toxicity, a combination therapy of levodopa and a potent therapeutic agent with a neuroprotective effect such as propolis is believed to provide a practical strategic option for long-term use.

Ayikobua et al., 2020 aimed to investigate the combined effect of propolis with levodopa in PTEN-induced putative kinase 1 (PINK1B9) mutant *Drosophila melanogaster* flies (*n* = 17 per group) (Ayikobua et al., 2020). Their results suggested that the treatment of propolis (500 mg/ml) and levodopa (250 mg/kg) combination for 21 days significantly improved the motor function as manifested as climbing activity, in which the improvement appeared to be higher than that of each monotherapy. In addition, strong anti-oxidant and hydrogen peroxide scavenging activities were detected in the propolis (500 mg/ml) monotherapy and combined propolis (500 mg/ml) and levodopa (250 mg/kg) treatment. Thus, it is speculated that the improved anti-oxidant activity in the combination group contributed to helping restore impaired tissue function in the mutant *Drosophila melanogaster* flies. Consequently, they have observed that propolis increased the life span across 93 days of mutant *Drosophila melanogaster* flies compared using levodopa only, although it was not as high as that in the propolis monotherapy and wild-type group, suggesting propolis may help lower the toxicity of levodopa in high dose. It is worth mentioning that there was no rigorous statistical analysis (i.e., CI) to support their claim of the synergistic activity of propolis and levodopa.

### Pharmacokinetic Drug–Herb Interaction With CYP450 Enzymes

Most medications administered in the body undergoing a chemical alteration primarily occur in the liver, namely, biotransformation as a way to create metabolites that are more easily excreted from the body (Saravanakumar, Sadighi, 2019). CYP450 enzymes are a group of hemeproteins essential for the biotransformation of medications in the liver (McDonnell and Dang, 2013). The activity of CYP450 enzymes is critical for the actual drug efficacy as it significantly affects the concentration of the drug in circulation and its metabolites. If the drug efficacy mainly relies on the original form (not its metabolites), the inhibition of its corresponding CYP450 enzyme activity leads to a reduced biotransformation activity and thus results in an increased concentration in the circulation and higher drug effects or even overdose-induced toxicities. In contrast, induction of CYP450 enzyme activities may result in a reduced drug effect and loss of efficacy (Zhou et al., 2021). Propolis is a mixture of a group of bioactive compounds mostly metabolized by the CYP450 family, and the effect on CYP450 has been increasingly characterized. Thus, it raises the concern of possible adverse events of combining propolis with various medications due to the changes in the activity of CYP450 enzymes. Table 3 summarizes the studies regarding the effects of propolis on CYP enzymes *in vitro* or *in vivo*.

**TABLE 3:** The effect of propolis on CYP450 *in vitro*, *in vivo,* and human.

Propolis samples	Subjects	Alteration on CYP450 enzymes	Key results	References
Propolis containing products (source and composition not specified)	Infected HepG2 cells with five P450-expressing adenoviruses (Ad-CYP1A2, Ad-CYP2C9, Ad-CYP2C19, Ad-CYP2D6, and Ad-CYP3A4)	↓ CYP1A2, CYP2C9, CYP2C19, CYP2D6, CYP3A4	The propolis containing product simultaneously inhibited CYP1A2, CYP2C9, CYP2C19, CYP2D6, CYP3A4 activities by more than 50%	Sasaki et al. (2017)
Ethanol extract of Brazilian green propolis (EEP-B55)	Human recombinant CYP1A2, CYP2C9, CYP2C19, CYP2D6, and CYP3A4 microsomes expressed in baculovirus-insect cells	↓CYP1A2, CYP2C9, CYP2C19, CYP2D6, and CYP3A4	EEP-B55 inhibited the activities of CYP1A2, CYP2C9, CYP2C19, CYP2D6, and CYP3A4 *in vitro* with IC_50_ values of 4.07, 2.62, 9.53, 18.9, and 20.6 μg/ml, respectively	Naramoto et al. (2014)
Propolis extract with characterized chemical composition	Human liver microsomes	↓CYP1A2, CYP2E1, and CYP2C19	Propolis extract inhibited CYP1A2, CYP2E1, and CYP2C19 with IC_50_ values of 6.9, 16.8, and 43.1 μg/ml. It showed no change on CYP2A6, CYP2B6, CYP2C9, CYP2D6, and CYP3A4	Ryu et al. (2016)
		No effect on CYP2A6, CYP2B6, CYP2C9, CYP2D6, and CYP3A4	In addition, the addition of propolis decreased the metabolites of duloxetine which is metabolized by CYP1A2 and CYP2D6, suggesting a possible drug–herb interaction of propolis and duloxetine	
Standardized propolis extract (EPP-AF^®^)	Healthy adult volunteers	No clinical change on CYP1A2, CYP2C9, CYP2C19, CYP2D6, CYP3A	EPP-AF^®^ did not show any clinical change on CYP1A2, CYP2C9, CYP2C19, CYP2D6, CYP3A activities, and the changes for AUC values of caffeine, losartan, omeprazole, metoprolol, midazolam, and fexofenadine were all below 20% when co-administered	Cusinato et al. (2019)

Sasaki et al. (2017) screened the 172 health foods on the activity levels of CYP1A2, CYP2C9, CYP2C19, CYP2D6, and CYP3A4 in human hepatocytes. In particular, a propolis-containing product was shown to inhibit all five CYP450s by more than 50%. However, the source, concentration, and chemical composition of this propolis-containing product were not specified in the study (Sasaki, Sato, 2017). A similar finding was shown by Naramoto et al. (2014), suggesting that a commercial ethanolic extract of the Brazilian green propolis (EEP-B55) inhibited these five CYP450s in baculovirus-insect cells with IC_50_ values ranging from 4.07 to 20.6 μg/ml (Naramoto, Kato, 2014). However, the results varied when propolis was tested in human liver microsomes, which showed that propolis extract (source not specified but chemical composition determined) only inhibited the activities of CYP1A2 and CYP2C19, and no effect was shown in CYP2A6, CYP2C9, CYP2D6, or CYP3A4. In addition, they have tested the activity in CYP2E1 and CYP2B6 with significant inhibition in CYP2E1 (IC_50_ = 16.8 μg/ml), which was not tested in any above-mentioned studies (Ryu, Oh, 2016). In order to unravel if the propolis extract is capable of affecting CYPs in the human body, Naramoto et al. (2014) investigated the blood concentrations of major bioactive compounds in propolis and examined if the concentrations were high enough to cause a clinical change of the CYPs in the body by propolis (Naramoto et al., 2014). Their results showed that artepillin C, kaempferide, dihydrokaempferide, isosakuranetin, and kaempferol contributed to the CYP450 inhibitory activity of a standardized propolis extract (EEP-B55) as the major bioactive compounds. Then, they investigated the blood concentration of these major bioactive compounds and compared that to the IC_50_ values against these CYPs in rats. Their results suggested that most of the bioactive compounds that showed CYP inhibition *in vitro* exhibited poor bioavailability even when EEP-B55 was administered fivefold of the recommended daily dose. Thus, their ability to inhibit CYPs in body was deemed insignificant due to their low amount in blood and hepatocytes. This assumption has been partially confirmed by Cusinato et al. (2019), showing that the effects of the propolis extract (standardized propolis extract: EPP-AF^®^) on CYP1A2, CYP2C9, CYP2C19, CYP2D6, and CYP3A were insignificant in healthy adult volunteers (Cusinato, Martinez, 2019). Furthermore, the change for AUC values of their corresponding prob drugs were all within 20%, which was considered not clinically significant. Thus, it was concluded from the human trial that propolis was safe regarding the potential interaction with CYP enzymes. However, it is worth mentioning that it is still essential to investigate the concentrations of the bioactive compounds in the propolis as a quality control procedure in order to estimate the effect on CYPs in the body due to the varied chemical composition of propolis collected from different sources and seasons.

## Conclusion and Future Perspective

In our current review, most of the pharmacodynamic studies suggested that propolis extract helped enhance the efficacy of anti-cancer, antibacterial, antifungal, anti-diabetic, anti-parasitic, anti-Alzhemic, and anti-Parkinson’s drugs. Enhanced activity with chemotherapies was conferred by greater anti-cancer effect, improved sensitivity to chemo-resistant tumors, and ameliorated side effects and toxicities induced by the chemotherapies. The enhanced anti-cancer activity was mostly attributed to the upregulation of the apoptotic pathway and downregulation of the NF-κB pathway. The reduced side effect was related to the activation of Nrf2-regulated antioxidant activity. Propolis also showed the ability to inhibit P-gp, which reduced chemoresistance *via* promoting the intracellular permeability of the chemotherapy.

Propolis, in combination with antibiotics and antifungals, contributed to an enhanced action in a reduced dose and showed better activity against resistant organisms. Thus, the combinations could be a better option to combat microbial resistance than using antimicrobial agents alone. The antibacterial activity was more significant against gram-positive bacteria than gram-negative, and better activity was reported in combination with antibiotics that interfere with cell wall integrity and protein synthesis. However, few studies have confirmed the molecular mechanisms for the enhanced antibacterial actions. The action of an anti-diabetic drug, metformin, was markedly enhanced by propolis extract through the protection of the pancreatic cells and major organs from oxidative damage with upregulated expression of the antioxidant proteins and downregulation of the inflammatory pathways. Propolis, in combination with anti-Alzhemic and anti-Parkinson’s drug donepezil and anti-Parkinsonism drug levodopa, showed better protection of the memory cells from oxidative damage and improved memory function and physiological parameters of PD along with the reduction of levodopa mediated side effects. Although several preclinical studies suggested the altered CYP450 enzymes with the co-incubation of propolis extract, the human trial demonstrated that the effect on CYP450 enzymes by propolis was insignificant due to the low bioavailability of contributing compounds presented in the propolis. The phytoconstituents of propolis can vary according to the source. Thus, the standardization of the chemical composition of propolis extract is essential to ensure the consistent quality and efficacy control of the propolis product.

By exploring the possible combinations of propolis with various types of drugs and related mechanistic actions, the knowledge provided in the present review is important for developing novel combination drug therapy in integrative medicine in the research area where the efficacy of conventional drugs is limited. However, it is worth mentioning that most of the interactions were demonstrated from *in vitro* and *in vivo* studies. They have not been validated in the clinical setting. In addition, many studies have overlooked the molecular mechanisms of the enhanced or protective effect, which require further attention. Although most of the studies aimed to investigate the possible synergistic effect of the combination therapies, they did not analyze or mention the combination index. Thus, it became difficult to justify the synergism and only revealed the enhanced or positive interactions.

Along with the above limitations, several areas require further scientific attention to completely describe the potential of propolis for the combination therapies. Particularly, the interaction between propolis and analgesic drugs (such as non-steroidal anti-inflammatory drugs) is because several of the above-mentioned studies have reported the downregulation of inflammatory pathways and cyclooxygenase enzymes in the combination therapies. Several studies have reported significant anti-viral, anti-hypertensive, lipid-lowering, neuroprotective, and neuroregulatory effects of propolis. Thus, the combination of propolis with the standard drugs used for the above diseases could be explored.

Taken together, propolis may interact with various pharmaceutical drugs, which may bring beneficial therapeutic outcomes and prevent unwanted clinical consequences. Thus, this review provides a comprehensive and quick reference for practitioners, consumers, and clinicians who need information on possible drug interactions with propolis.
