# Supplementary material for: An Overview of the Evidence and Mechanism of Drug–Herb Interactions Between Propolis and Pharmaceutical Drugs
Source: Front Pharmacol. 2022 Apr 4;13:876183. doi: 10.3389/fphar.2022.876183 (PMC9015648; doi:10.3389/fphar.2022.876183)
Supplement: Supplementary file 1 [file Image1.pdf]

Supplementary Material

1.1 Supplementary Figures

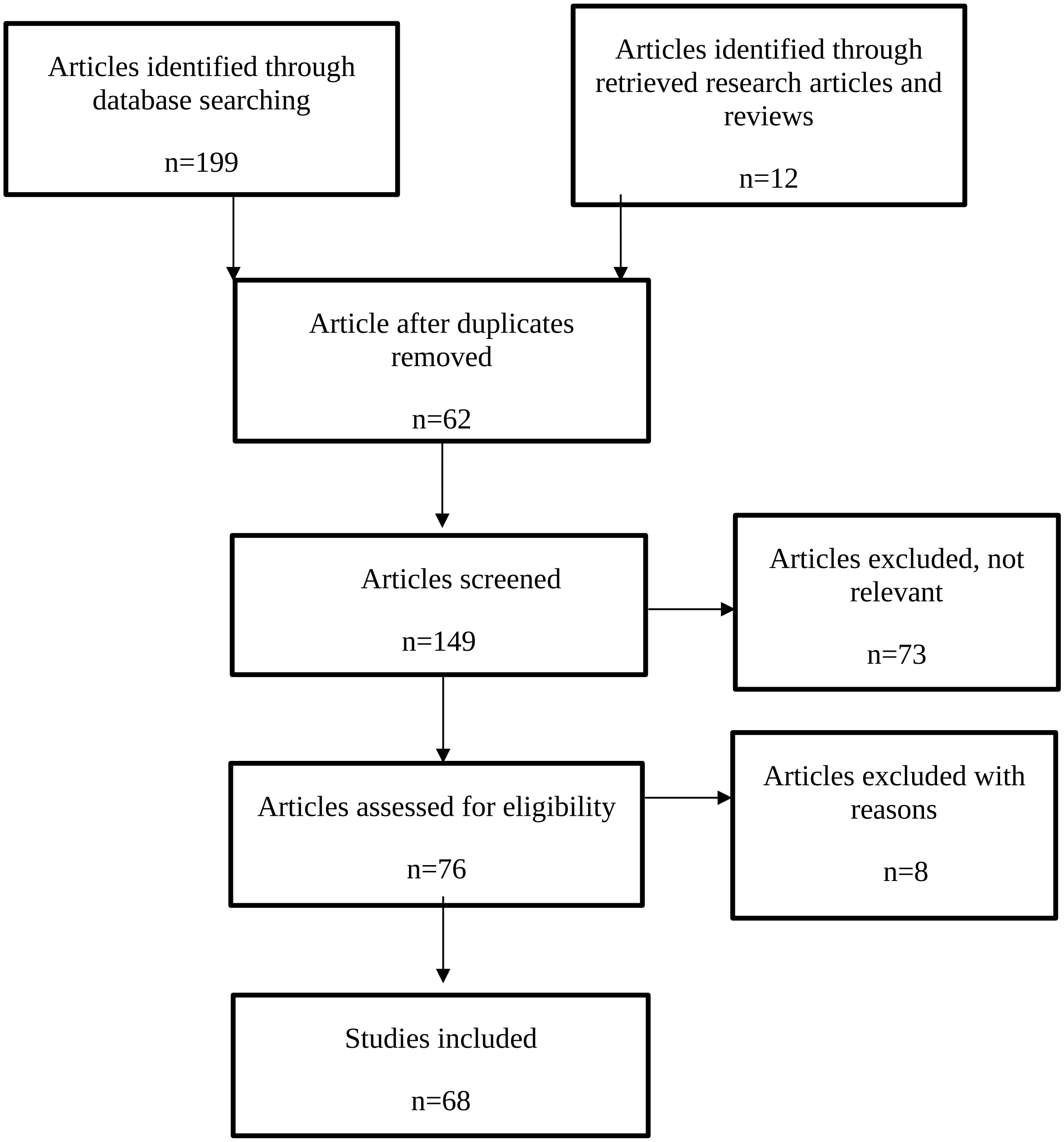

Supplementary Figure 1. Flow chart of the study selection process.
